# Diversity and metabolic energy in bacteria

**DOI:** 10.1093/femsle/fnad043

**Published:** 2023-05-16

**Authors:** Ben Allen, Rebeca Gonzalez-Cabaleiro, Irina Dana Ofiteru, Lise Øvreås, William T Sloan, Donna Swan, Thomas Curtis

**Affiliations:** School of Engineering Newcastle University, Newcastle upon Tyne NE1 7RU, UK; Department of Biotechnology, Delft Technical University, Postbus 5, 2600 AA Delft, The Netherlands; School of Engineering Newcastle University, Newcastle upon Tyne NE1 7RU, UK; Department of Biological Sciences, University of Bergen, Postboks 7803 5020 Bergen, Norway; Department of Civil Engineering, Glasgow University, Glasgow G12 8QQ, UK; School of Engineering Newcastle University, Newcastle upon Tyne NE1 7RU, UK; School of Engineering Newcastle University, Newcastle upon Tyne NE1 7RU, UK

**Keywords:** diversity, metabolic, energy, bacteria

## Abstract

Why are some groups of bacteria more diverse than others? We hypothesize that the metabolic energy available to a bacterial functional group (a biogeochemical group or ‘guild’) has a role in such a group’s taxonomic diversity. We tested this hypothesis by looking at the metacommunity diversity of functional groups in multiple biomes. We observed a positive correlation between estimates of a functional group’s diversity and their metabolic energy yield. Moreover, the slope of that relationship was similar in all biomes. These findings could imply the existence of a universal mechanism controlling the diversity of all functional groups in all biomes in the same way. We consider a variety of possible explanations from the classical (environmental variation) to the ‘non-Darwinian’ (a drift barrier effect). Unfortunately, these explanations are not mutually exclusive, and a deeper understanding of the ultimate cause(s) of bacterial diversity will require us to determine if and how the key parameters in population genetics (effective population size, mutation rate, and selective gradients) vary between functional groups and with environmental conditions: this is a difficult task.

## Teaser

The diversity of a group of bacteria appears to be related to the energy it can gain from the chemicals it uses to drive its metabolism.

## Introduction

Universal patterns of diversity have played a strategically important role in classical ecology (Rosenzweig 1995), and microbial ecologists have sought, with vary degrees of success, to observe such patterns in microbial communities (Bell et al. 2005, Horner-Devine et al. 2004). We report a ubiquitous pattern in the diversity of bacterial functional groups.

A functional group (also called a guild or biogeochemical group) is a collection of bacteria that exploit the same redox couples. For example, ammonia and oxygen (ammonia-oxidizing bacteria) or glucose and sulphate (sulphate-reducing bacteria). We hypothesize that the energetic yield of a particular redox couple, directly or indirectly, controls the genetic diversity of organisms belonging to the corresponding functional group.

There are multiple reasons to suspect that energetic yield (as opposed to the energy impinging on an environment) plays a role in differences in diversity (Curtis et al. 2008). Not only is energy one of the few concepts to have genuine predictive power in microbial ecology (Broda 1977, Brown 2000, Ettwig et al. 2010, Harte and Newman 2014, Harte et al. 2008, Jetten et al. 1998), it is also held to underlie many macroecological patterns in classical ecology (Brown 2000, Harte and Newman 2014, Harte et al. 2008) and local diversity in hot springs (Dick and Shock 2013). Evolution is implicitly about the processing of information (Kimura 1961), and there is a link between energy and information in biology (Wolpert 2016, Kempes et al. 2017). Energy has also been hypothetically linked to the evolution of higher organisms (Lane and Martin 2010), though that reasoning has been challenged (Lynch and Marinov 2016).

To test this hypothesis, we must estimate three things: the metabolic energy available to a member of a given functional group, the extent of bacterial diversity, and the assignment of taxa to functional groups.

The first, the energetic yield of a cell, can be roughly modelled from first principles (Rittmann and McCarty 2001). For any given functional group, the catabolic energy available to the cell (∆G_cat_) is calculated from the free energies of the redox couples exploited. Some of this energy is required for anabolism (∆G_ana_ kJ/mole biomass formed) (much more if carbon is fixed), some for cellular maintenance, and some dissipated (∆G_dis_) during biomass synthesis. The maximum growth yield is a function of catabolism, anabolism, and dissipation (Maximum Yield = ∆G_cat_/(∆G_ana_+ ∆G_dis_) (Kleerebezem and Van Loosdrecht 2010) (Table 1). This is only an approximation because: (1) we must assume a single electron donor, (2) the actual energy depends on the environmental conditions (pH, solute concentration, temperature, and pressure), and (3) we neglect maintenance energy. However, we posit that this approximation is ‘good enough’ to test our hypothesis because we are looking at the differences over nearly 3 orders of magnitude (Table 1). Thus, though many factors could impinge on this relationship, they are unlikely to obscure a strong and consistent correlation, should it exist. For example, differences in environmental conditions will impact on free energy from any given redox couple. However, the effects are linear (driven by temperature and the natural log of the ratio of the reactants and products) and modest when compared to the very large differences between redox couples. Likewise, most monomeric sugars or amino acids would have similar (but lower) free energies to one we have assumed. Maintenance energy, which we have neglected, is of the order of 1–10 kJ/mole biomass and has a negligible (median <1%) impact on the calculated yield in cultured heterotrophs. Maintenance energy is more important in organism lower energy redox couples, but maintenance energy ‘in the wild’ is thought to 3–6 orders of magnitude lower than the classical Tijhuis data.

**Table 1.** The metabolic energies used to calculate the energetic or growth yield.

Metabolism	DG_cat_ (kJmol^−1^)	DG_anab_ (kJmol^−1^)	DG_dis_ (kJmol^−1^)	Growth yield
ANAMOX	−363	−43	3500	0.10
Acetoclastic methanogen	−31	30	432	0.07
Aerobic heterotroph	−2841	−24	236	13.42
Ammonia oxidizer	−283	274	3500	0.07
Methylotrophic methanogen	−79	−33	651	0.13
Iron III reducer	−809	30	1088	0.72
Fermenting heterotroph	−204	−29	236	0.99
Hydrogen oxidizing methanogen	−34	−25	1088	0.03
Hydrogen oxidizer	−237	−25	1088	0.22
Methanotroph	−813	46	986	1.26
Methylotroph (methanol)	−689	−216	986	0.89
Nitrite oxidizer	−79	313	3500	0.02
Sulphur oxidizer	−797	55	1088	0.70
Sulphate reducer (complete ox)	−100	26	377	0.25
Sulphate reducer (H_2_)	−38	−25	1088	0.04
Sulphate reducer (partial ox to acetate)	−14	26	377	0.03

The values obtained depend on the stoichiometries employed, which are shown in the supplementary methods. ΔG_cat_ is the energy released by the redox couple expressed per mole of electron donor. ΔG_anab_ is the anabolic energy required to generate a mole of biomass from representative substrates for a given metabolism and may endergonic or exergonic. ΔG_dis_ represents the energy dissipated in anabolism and catabolism.

The second, the diversity of a bacterial community, is usually determined from the variation and proportional abundance of a ubiquitous and conserved molecular marker, typically the gene for the 16S RNA molecule. Bacterial diversity is often thought of as having several dimensions: the two most prominent being the absolute number and distribution (‘evenness’) of the taxa. The practical and theoretical barriers to determining the absolute diversity or the true distribution are formidable. Consequently, microbial ecologists use a wide range of imperfect indices and estimators instead. We have chosen a robust estimator that is theoretically suited to the hypothesis we are addressing. In practice, diversity measures are typically highly correlated (Walters and Martiny 2020).

The third thing we must infer, which taxa belong to which functional group, is the most problematic. The basic concept is simple one must compare the taxa—in our case, the genus—observed in an environment with a database [such as KEGG (Kanehisa et al. 2023)] of organisms of known function. There are a number of tools for doing this (Wemheuer et al. 2020). Errors will occur, for example, if the databases are imperfect (which is inevitable, all databases are biased towards cultured organisms) or the taxon observed has no known function (this is also inevitable, especially for less well-studied functions). Furthermore, some judgement is required when using a database like KEGG to infer function. For example, KEGG reports that the canonical ammonia oxidizer *Nitrosomonas europaea* has genes for dissimilatory sulphate reduction. The best results are probably best obtained when the reference database is calibrated for a given environment, but this may not be appropriate when, as in this study, multiple environments are considered. We therefore elected to categorize our genera ‘by hand’ using Bergey’s manual (Whitman et al. 2015).

Obviously, the effect of an error in any one of these three areas would be to obscure the hypothesized relationship between metabolic energy and bacterial diversity. Thus, if we are able to discern an energy-diversity relationship, that relationship is probably a robust one.

We tested the hypothesized energy-diversity relationship using classical methods to determine energetics, the 16S RNA gene to evaluate diversity, and a simple classification method to infer function.

## Methods

### Assignment to functional groups

Functional group assignments were determined initially by reference to Bergey’s Manual of Systematic Bacteriology (Whitman et al. 2015), followed by multiple rounds of manual curation. All members of a genus are assigned the same metabolism, and those for which a single classification would be unrealistic have been removed from the analysis. In all 964 genera were classified, and the classifications can be inspected on the Github page (https://github.com/beadyallen/EnergyEvoRate) in the ‘Intro’ file.

Facultatively, aerobic fermenters have, for the purposes of this study, been grouped along with aerobic heterotrophs. We recognize that in doing so, there is a danger of undue weighting of classifications in favour of higher energies, which would affect the balance between aerobic heterotrophs (over-representation) and fermenters (under-representation). Furthermore, we have elected to exclude the large prokaryotic group of organisms capable of phototrophic growth, on the grounds that the energy model employed does not readily extend to photosynthesis. We envisage that extensions to this work would include more detailed classification of a taxa’s metabolic category through reference to metabolic pathway databases.

### Energetics

Free energies of anabolism and catabolism were calculated (for pH, 298.15 K, 1 M, and 1 bar) using the methods described previously (Gonzalez-Cabaleiro et al. 2015). The Python code and values used are online (https://github.com/beadyallen/EnergyEvoRate under the metabolics tab). The value of ΔG_cat_ is given by the change in free energy between reactants and products in the relevant complete catabolic reaction, per mole of electron donor. Aerobic heterotrophs are assumed to process a complete glycolysis to tricarboxylic acid cycle conversion to CO_2_ and H_2_O, while anaerobic fermenters are assumed to use glucose as the primary substrate in a mixed acid fermentation pathway with acetate and H_2_ being produced in stoichiometric quantities. We have also determined the yields for homolactic, heterolactic, and Stickland-type fermentations and found all values to be similar. While an alternative choice of fermentation pathway for a particular taxon would lead to slightly different results, we do not believe the overall results of this study will be altered. Given the difficulty in reliably assigning a single functional group at the genus level, we maintain our approach is a ‘good enough’ approximation.

Anabolism is calculated as a combination of both ‘true’ anabolic free energy (per mole of new biomass), ΔG_ana_, and ΔG_dis_—the energy dissipated to the surroundings. For the calculation of ΔG_ana_, the unit of biomass is taken to be CH_1.8_O_0.5_N_0.2_ (Roels 1983). ΔG_dis_ is calculated using the Gibbs Dissipation Method (Heijnen et al. 1992).

The maximum energetic was calculated and expressed as a ratio.


$$
\begin{eqnarray*}
Maximum\,\, energetic\,\, yield = \frac{{{\rm{\Delta }}{G}_{cat}}}{{{\rm{\Delta }}{{\rm{G}}}_{ana} + {\rm{\Delta }}{G}_{dis}}}
\end{eqnarray*}
$$

Our approach is necessarily simplified and idealized for two reasons. Firstly, standard physiological conditions will not necessarily represent the conditions in nature either now or over evolutionary time, and secondly, a single representative organic carbon source (glucose) was used in place of the unknown and unknowable range of organic matter bacteria may consume.

To partially validate our estimates of yield, the modelled yield for aerobic heterotrophs (13.1 j/j) was compared with the empirical yields reported by DeLong et al. (2010).

De-Long et al. (2010) recorded the maximum specific growth rate in reciprocal time (d^−1^), wet mass (g), and metabolic rate (W) for 33 cultured bacterial species growing on glucose. We calculated the specific substrate uptake rate by dividing the active (as opposed to basal) metabolic rate of the cell Watts (Js^−1^) by the mass (g) and converting the time unit from seconds to days. On this basis, we could determine the energetic yield (gJ^−1^) by dividing the maximum specific growth rate by the specific substrate rate. De-Long made an identical calculation and used the term efficiency of biomass production for energetic yield. To convert the yield from g per joule to joules per joule, we multiplied by 0.2 (to allow for dry weight) (Makarieva et al. 2005) and assumed 22 523 joules per gramme of dry weight (Prochazk et al. 1970) (originally 5383  cal/g). The yields are lognormally distributed, and the geometric mean value of 13.47 j/j of the De-Long data are very close to the theoretical value (13.1 j/j) for yield on glucose (Supplementary Fig. S1).

### Data sources

The projects were selected from source biomes in the European Bioinformatics Institute MGnify (https://www.ebi.ac.uk/metagenomics/), Operational Taxonomic Unit tables were downloaded and extracted using a Python script (https://github.com/beadyallen/EnergyEvoRate). At the time of retrieval, all EBI projects were generated by version 2 of the EBI metagenomics portal. Local sample collections were processed through a standard QIIME pipeline (1.9.1) with open reference OTU picking according to 97% similarity with the Green Genes database (13.5) and classified against a database derived from Bergey’s manual, the database is to be found here: (https://github.com/beadyallen/EnergyEvoRate).

The sources for the biomes were as follows. For humans: a citizen science survey of the human microbiome (American gut ERP012803) (McDonald et al. 2018) and from malnourished Malawian children (Malawian children ERP005437) (Kau et al. 2015). For marine: a latitudinal transect of the Atlantic Ocean with a depth range from 20 to 200 m (Atlantic, ERP012887) (Milici et al. 2016) and Baltic Sea Microcosms from the Leibniz Institute for Baltic Sea Research BIOACID programme (Marine ERP013553) (Bergen et al. 2016). For freshwater: 386 Canadian Freshwater Bodies [Canada Water ERP012927 (Niño-García et al. 2016)] and water troughs on duck farms (DuckWater ERP012631) (Schenk et al. 2016). For wastewater: anaerobic digesters in England and Scotland (Donna_sludge ERP123489) and biomass from the biological stage of seventeen activated sludge wastewater treatment plants in the Severn Trent region, in the UK (Severn_Trent ERP123488) (DNA extracted using the MPBIO fast kit (MP Biomedicals, UK) and analysed using amplicon-sequencing of evolutionary-conserved 16 rRNA gene fragments on the MiSeq Illumina platform, (Caporaso et al. 2011). For soils: irrigated soils in Mexico (Mexican Soil SRP037963 (Broszat et al. 2014) and from Ny Alesund in Svalbard (Lise Soils ERP123490) (DNA was isolated from 0.5 g of soil samples using the BIO101 FastDNA Spin Kit (for soil; Q-Biogene, MP Biomedicals, UK), and 1 μl was taken and amplified as described by Quince et al. (2009), but with only 23 cycles in the PCR. The PCR amplicons (tags), were pooled and sequenced by 454 pyrosequencing on a Roche GSFLX system Quince et al. (2009). Volcanic: from Favara Grande in the Mediterranean (Volcanic ERP010094) (Gagliano et al. 2016) and hydrothermal vents from the subsurface of the Indian Ocean (Hydrothermal ERP011826) (Han et al. 2018). The joining of a hydrothermal vent and a volcanic soil was a mistake (see no ‘cherry picking’ below’). The Tara Ocean Survey (Tara ERP001736) (Pesant et al. 2015) was used as an independent sample.

To avoid ‘cherry picking’, an EBI 16S dataset, once chosen, could not be withdrawn from the study. However, an attempt to use the GreenGenes database (DeSantis et al. 2006) to evaluate the phylogenetic diversity has been recorded in supplementary methods (Supplementary Figs. S3 and S4). We are not relying on this data to support our argument because an informal reviewer pointed out the database was out of date, and we (subsequently) realized that such databases are biased towards cultured organism so are not random samples of the diversity of all functional groups.

### Diversity estimation

All data analysis was performed using R (v3.3.1), and detailed scripts are available for download at (https://github.com/beadyallen/EnergyEvoRate). Briefly, alpha diversity (inverse Simpson’s Index) was determined using the estimate richness function in the vegan package. Metacommunity diversity (*θ*) was used as a proxy for gamma diversity across metabolic groups and determined for each metabolic group using NMGS code (Harris et al. 2017) (https://github.com/beadyallen/EnergyEvoRate). This method calculates the size of the metacommunity that would be required to explain the diversity observed locally. The *θ * is a compound parameter and is (theoretically) equal to the number of individuals in the metacommunity multiplied by 2 times the speciation rate. We do not assume that communities are neutrally assembled. Other diversity measures can be chosen using this software and give comparable results. The complete GreenGenes 13.5 annotated OTU tree was pruned using functions from the ape package to include only genera relevant to our analysis, leaving 189 743 tips. Phylogenetic diversity was determined as the sum of branch lengths within each metabolic group. The data for phylogenetic distance (in the GreenGenes dataset), yield, and catabolism were subject to Box-Cox transformations, and a natural log transformation was selected. The energetic yield data used with the GreenGenes data was slightly better supported by a *X*^−0.5^ transformation, but the natural log transformation was preferred for simplicity and clarity and still gave normally distributed residuals after regression against phylogenetic distance.

## Results

The catabolic, anabolic, and dissipated energies were determined for each redox couple (under standard physiological conditions) and used to calculate the energetic yield (Table 1). Catabolic energy accounts for most of the differences in energetic yield between functional groups. The model is necessarily a generalization that neglects many important metabolic details. Moreover, the relative importance of these other factors will increase as the energy available to the cell decreases. But the comparison with the DeLong data (S1) sets suggests we have correctly estimated the median yields and that the other factors are incorporated into the normally distributed noise.

The diversity of individual metabolic groups in each biome was calculated using both the Inverse Simpson’s diversity index and metacommunity diversity (*θ*), with rankings of diversity across metabolic groups being consistent across biome types (Friedman’s test *P* = 9.3 × 10^−5^).

**Figure 1.**
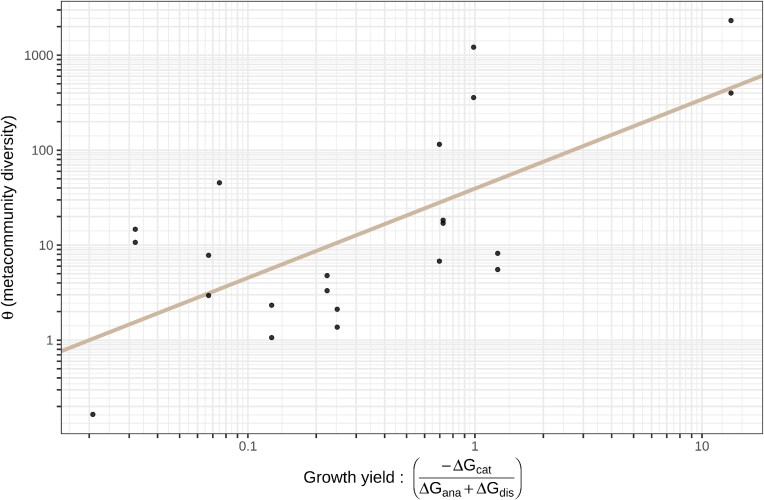
The relationship between the estimated metacommunity diversity and the energetic yield in wastewater treatment plants in the United Kingdom. *P*-value of slope = 0.0004, R^2^ = 0.443.

We initially examined the relationship between the estimated energetic yield and metacommunity diversity in wastewater treatment plants (Fig. 1). Intrigued by the relationship we found, we sought to compare this finding with other biomes to see if it was observed more widely; it was.

**Figure 2.**
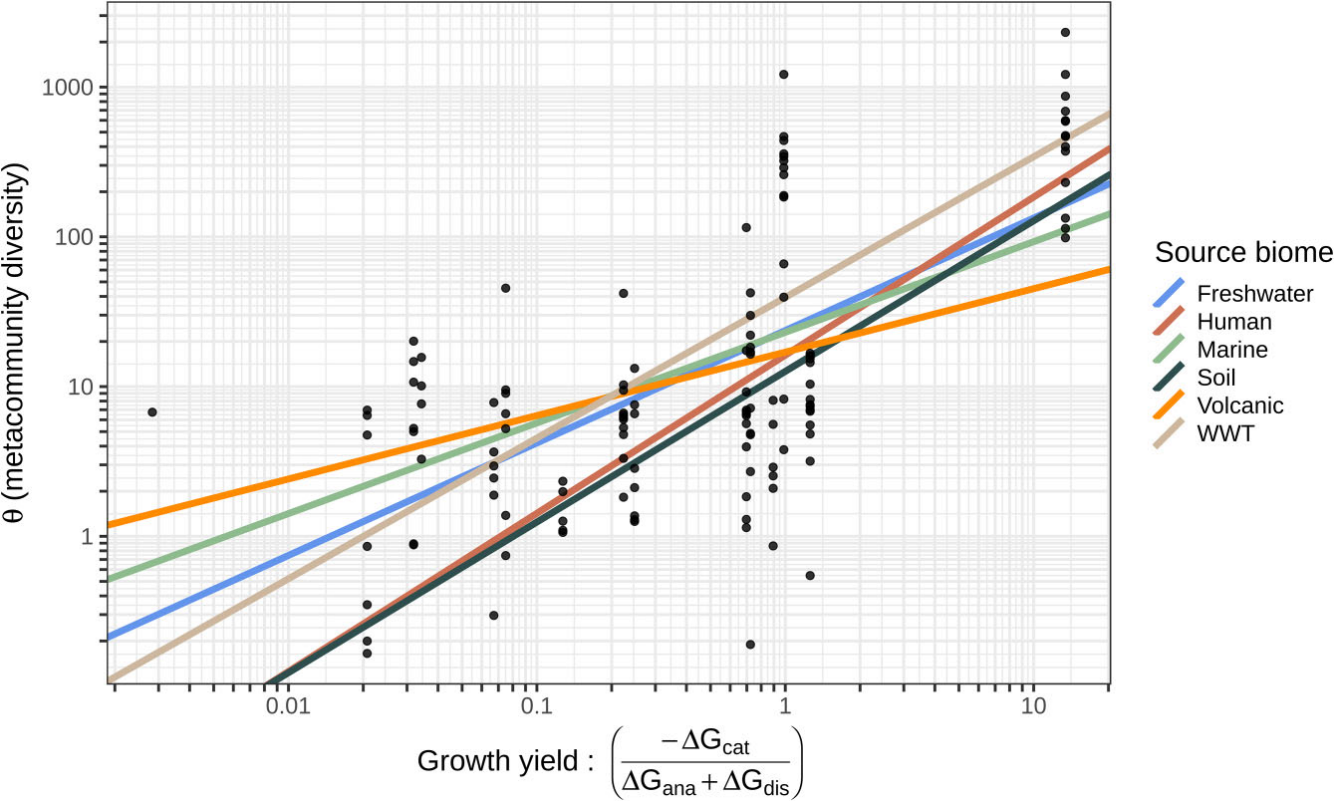
The relationship between the estimated metacommunity diversity in all biomes and the energetic yield. The overall slope is significant (slope = 0.73, *P* slope < 0.001; adjusted R^2^ = 40%). Values for individual biomes are given in Table 2.

There was a good relationship (Fig. 2) between the estimated energetic yield and metacommunity diversity in all biomes (slope 0.73, *P *< 0.001; adjusted R^2^ = 40%), with residuals being normally distributed.

The energy-diversity relationship was significant for all the individual biomes with the exception of the volcanic dataset, where we had accidentally mixed a deep-sea hydrothermal vent with a soil (Table 2). The range of slopes was a relatively narrow (∼0.6–1).

**Table 2.** The slopes (and associated statistics) for the relationship between metacommunity diversity and energetic yield.

Biome	Slope	*P*_(slope)_	Intercept	*P*_(int)_
Freshwater	0.75	2.19E-04	3.16	1.98E-08
Human	1.06	9.24E-03	2.79	5.35E-04
Marine	0.61	2.88E-04	3.14	1.50E-11
Soil	1.01	2.40E-06	2.54	7.68E-09
Volcanic	0.42	8.86E-02	2.83	1.67E-04
WWT	0.94	4.28E-04	3.67	9.65E-08

The median value for the significant slopes is 0.95.

Analogous results can be found by comparing the different diversity measures with the catabolic energy (∆G_cat_) (Supplementary Fig. S2). Moreover, though metacommunity diversity was chosen as the most appropriate measure of diversity, other measures of ‘richness’ can be examined using the software.

We considered that some, or all, of the observed energy-diversity relationship could prosaically be attributed to the greater metabolic versatility of functional groups guilds with a range of complex substrates (aerobic heterotrophs, fermenting organisms, sulphate reducers, and iron reducers).

**Figure 3.**
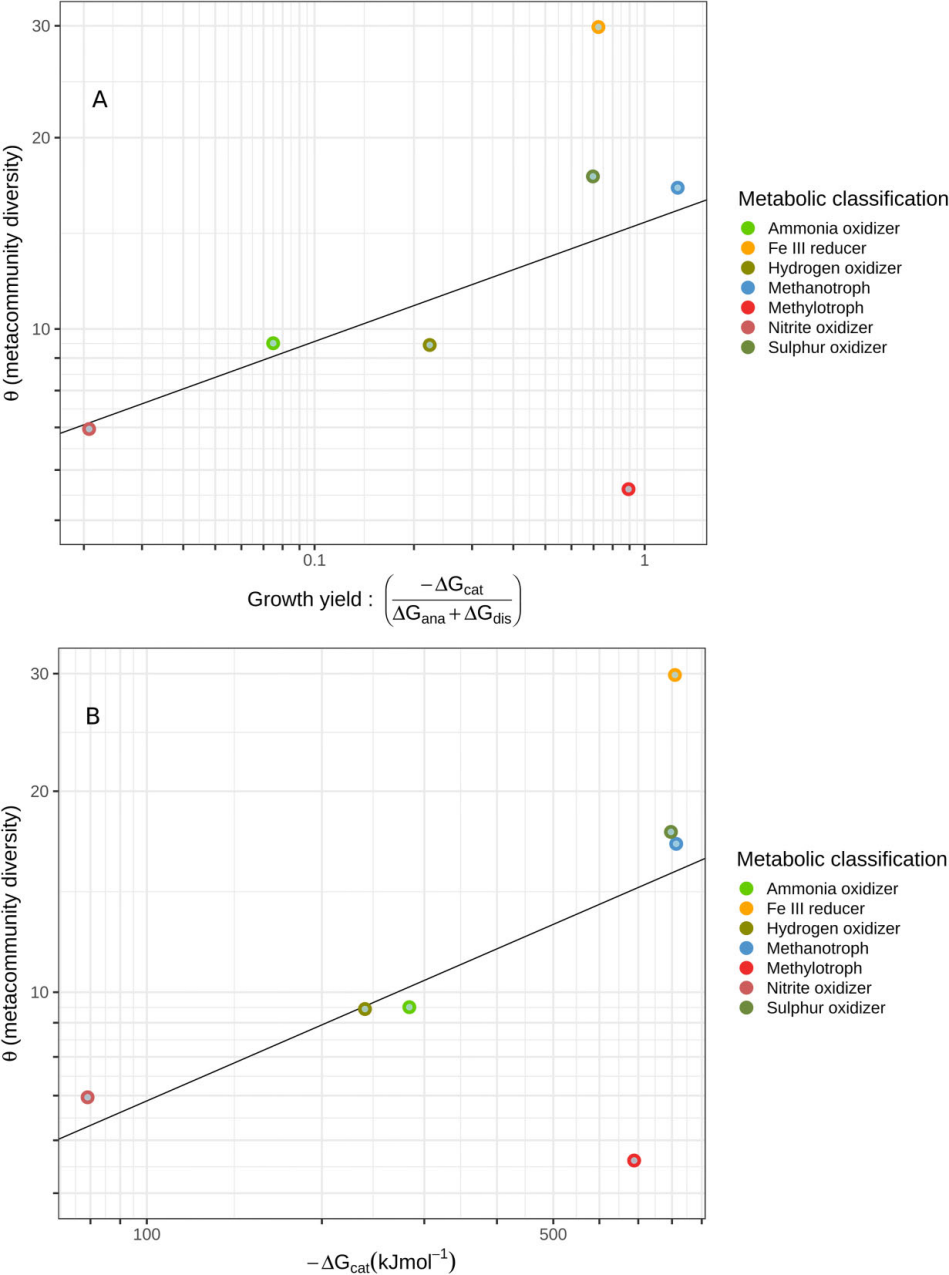
The relationship between the meta-community diversity of functional groups in the EBI-Tara dataset thought to have simple substrates and: (A) energetic yield (not significant) (B) catabolic energy a (*P* = 0.02, R^2^ = 79%).

We therefore analysed the relationship between energy and diversity, examining those functional groups purported to have simple (typically a single) substrates. A new dataset was chosen (the EBI-Tara Oceans) (Sunagawa et al. 2015). The relationship between energetic yield and the metacommunity diversity of functional groups with simple substrates was not statistically significant (Supplementary Fig. 3A). However, a credible (*P* = 0.02, R^2^ = 76%) relationship was found between catabolic energy and metacommunity diversity (Fig. 3B).

Methylotrophs were an obvious outlier. Our analysis distinguishes between the true methanotrophs (genera that, as a first step, oxidize methane to methanol) and methylotrophs (those deriving energy from extracellular methyl compounds such as methanol). It could be argued that combining into a single guild makes the most sense. However, since *post*-*hoc* removal of methylotrophs from the analysis could be construed as ‘*P*-hacking’, we have elected to retain a separate methylotroph classification.

## Discussion

We conclude that there are universal and systematic differences in the diversity of functional groups (on the basis of the consistent ranking in diversity) and that these differences are directly or indirectly linked to the metabolic energy of the cell (on the basis of the observed correlations between energetic yield and diversity). This energy-diversity relationship is, potentially at least, analogous to the patterns in diversity and space seen in classical ecology. Such ubiquitous patterns are imbued with significance because they tell ‘us that diversity is a predictable variable susceptible to scientific analysis’ (Rosenzweig 1995).

We have considered the alternative interpretation: that the putative energy relationship was simply attributable to the elevated diversity of the heterotrophs and variation in the diversity of the low-energy taxa is just noise. The apparent persistence of our proposed pattern in a separate ‘single resource’ marine data set (from the TARA study) suggests there is an energy pattern in the non-heterotrophic taxa. Finally, we attach particular significance to the repeatability of the overall pattern across biomes and data sets and the relative consistency of the energy-diversity slopes within biomes.

We note that this pattern was discerned even though our methodology had many imperfections. Our study was potentially limited by: the number of functional groups for which we can propose a redox couple, the approximations of free energies, and difficulties in the attribution of function and the estimation diversity (Pielou 1977) (Suzuki and Giovannoni 1996). We hope that subsequent studies, with improved methodologies, could yield an even better picture of the relationship between metabolic energy and diversity.

However, the true benefit of a finding a consistent pattern in nature comes not from identifying the pattern, but from the search for, and discovery of the underlying mechanism or mechanisms. Consequently, it would seem sensible to ask what mechanisms might underlie the apparently ubiquitous relationship between metabolic energy and bacterial diversity.

Classical ecological theory suggests that environmental variation plays a central role in the generation (Levins 1968) and maintenance of diversity (Tilman 1982). Microbial ecologists typically concur (Cohan 2017). If environmental variation explains our findings, then there is an energetic limit on the amount of environmental variation and thus the number of ecotypes or niches. This is easy to conceive for functional groups using organic matter as an electron donor (aerobic heterotrophs, fermenter, iron-reducing bacteria, and sulphate-reducing bacteria), where the breadth of viable organic electron donors and end products (Großkopf and Soyer 2016), and thus the range of ecotypes, could be dictated by the redox value of the electron acceptor. For example, some substrates are only energetically viable in the presence of oxygen. For bacteria with single substrates the energy could still affect the range of ecotypes by placing constraints on the range of energetically demanding environments that are viable (Oren 2011).

If variation did scale with energy, there could be a signal in the mutation rates of the bacteria. A number of theoreticians (using a variety of simplifying assumptions) have proposed that the rate of mutation will come into balance with the rate of selection or environmental variation (Dawson 1998, Gillespie 1981, Kimura 1967, Leigh 1970, Orr 2000, Sturtevant 1937). Though there is only circumstantial evidence for this proposal (Drake et al. 1998). But if the theories were correct and environmental variation did underlie our observations, we would expect mutation rates to scale positively with metabolic energy.

We think it is instructive to at least consider other explanations. Not least because attempts to experimentally demonstrate a relationship between bacterial diversity and environmental variation have previously failed in both communities (Pholchan et al. 2010) and experimental bacterial populations (Jasmin and Kassen 2007).

A very simple explanation is that the number of generations controls diversity, and generation times could be affected by metabolic energy. However, the actual number of generations will depend on the amount of redox donor and acceptor available (all of which can vary between biomes) and the age of the lineage (some of the oldest lineages are the least diverse). More generally, generation time does not appear to be the universal and consistent mechanism required to explain our observations (Gibson and Eyre-Walker 2019).

Others have previously conjectured that mutational load (which has an absolute as opposed to relative effect on growth rate) would be felt more acutely by bacteria with less available energy (Curtis et al. 2008). However, in classical population genetics, the mutational load is dictated by the rate of mutation and not the deleterious effect of the mutations *per se* (Haldane 1937). Though dynamic models of the evolution of mutation have found that the deleterious effect of a mutation can influence mutation rate, the effect is very modest and only apparent at certain parameter values (Andre and Godelle 2006, Johnson 1999). In any case, diversity reflects the fixation of mutations, not simply the rate at which they are generated.

The small but unavoidable energetic cost of the loss of entropy that information storage entails could be responsible for the differences in diversity we have observed. In principle, the costs of entropy do place an absolute upper limit on the rate of information processing in a cell and a microbiome (Wolpert 2016, Kempes et al. 2017). In practice, the costs—though not well understood—appear to be very small indeed, and so we, for the time being, discount this mechanism.

A simple and testable explanation is also offered by the drift barrier: only alleles with a selection coefficient greater than the power of drift *(∼1/N_e_)* where *N_e_* is the effective population size) are subject to selection and thus fixation (Lynch 2012, Lynch et al. 2016, Sung et al. 2012). If the metabolic energy affected either the effective population size or the relative impact of selection on fitness, this could lead to a relationship between diversity and energy.

It is difficult to distinguish between these explanations. The proposed mechanisms are not mutually exclusive. The generation of bacterial diversity is perhaps subject to a hierarchy of controls. For example, environmental variation (∼niches) but could be important in generating diversity, but within a limit set by the drift barrier. To gain a deeper understanding, we need to determine how the fundamentals of population genetics (effective population size, mutation rates, and selection coefficients) vary between functional groups and with environmental conditions. Determining these fundamentals will not be easy, but the quantitative description of determinants of, and limits to, natural selection in microorganisms is a worthwhile endeavour (Gould and Lewontin 1979).

## Supplementary Material

fnad043_Supplemental_File

## Data Availability

All the data are in publicly available databases and the code may be accessed through the Github using the link embedded in the methods.
