# Supplementary material for: Diversity and metabolic energy in bacteria
Source: FEMS Microbiol Lett. 2023 May 16;370:fnad043. doi: 10.1093/femsle/fnad043 (PMC10214464; doi:10.1093/femsle/fnad043)
Supplement: fnad043_Supplemental_File [file fnad043_supplemental_file.docx]

Supplementary Methods

Diversity and Metabolic Energy in Bacteria

Ben Allen^1^, Rebeca Gonzalez-Cabaleiro^4^, Irina Ofiteru^1^, Lise Øvreås^3^, William Sloan^2^, Donna Swann^1^, Thomas P Curtis^*1^

^1^School of Engineering Newcastle University, Newcastle, NE1 7RU, UK

^2^Department of Civil Engineering, Glasgow University, Glasgow, G12 8QQ, UK.

^3^Department of Biological Sciences, University of Bergen, Postboks 7803

5020 Bergen, Norway.

^4^Department of Biotechnology, Delft Technical University, Postbus 52600 AA Delft, The Netherlands


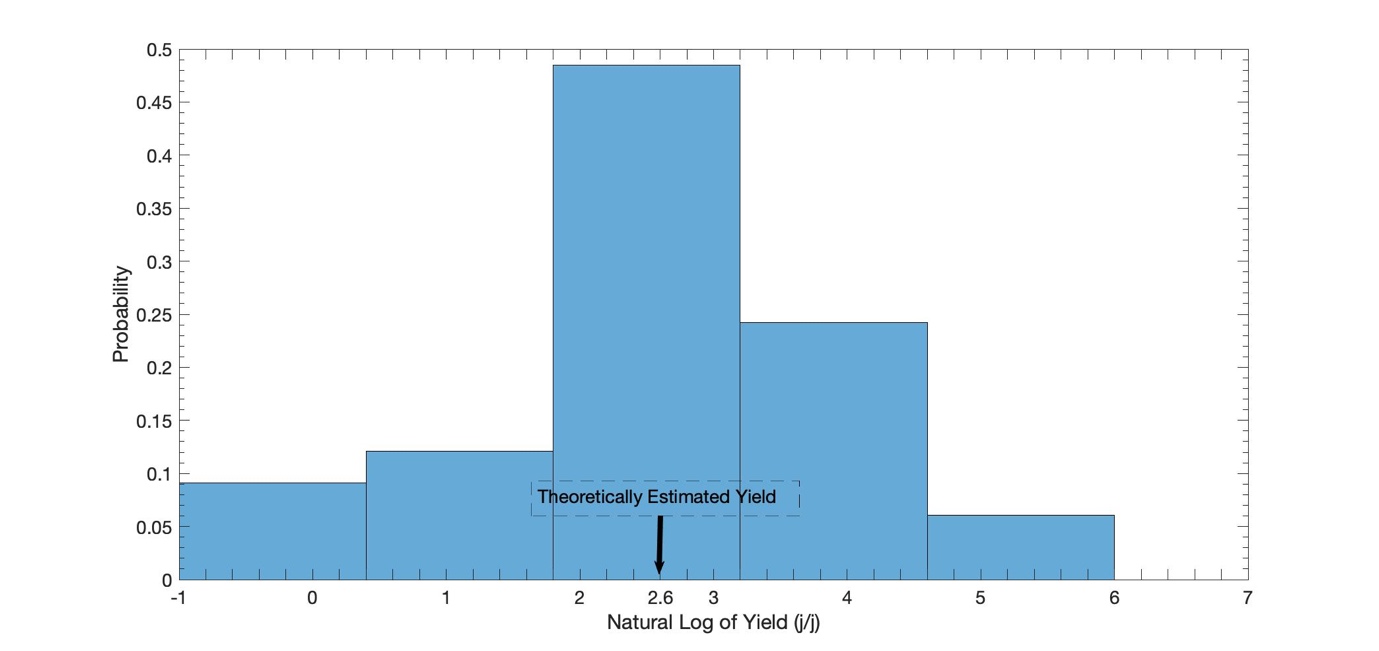


Figure S1 The relationship between our estimate of energetic yield (joules/joule) and the yield observed in in heterotrophic bacteria from the DeLong et al., dataset (DeLong, et al. 2010: 12941-5). De-Long et al (DeLong, et al. 2010: 12941-5) recorded the maximum specific growth rate in reciprocal time (d^-1^), wet mass (g) and metabolic rate (W) for 33 cultured bacterial species. We calculated the specific substrate uptake rate by dividing the active metabolic rate of the cell Watts (Js^-1^) by the mass (g) and converting the time from seconds to days. On this basis we could determine the energetic yield (gJ^-1^) by dividing the maximum specific growth rate by the specific substrate rate. To convert the yield from g per joule to joules per joule we multiplied by 0.2 , to allow for dry weight (Makarieva, et al. 2005: 2219-24), and assumed 22,523 Jg^-1^ of dry weight (Prochazk.Gj, et al. 1970: 646-&) this gives a geometric mean value of 13.47 j/j. All datasets were examined using a Box-Cox transformation and a lognormal transformation chosen. All statistical analysis was undertaken in the Matlab R2017a (Natick, MA, USA).


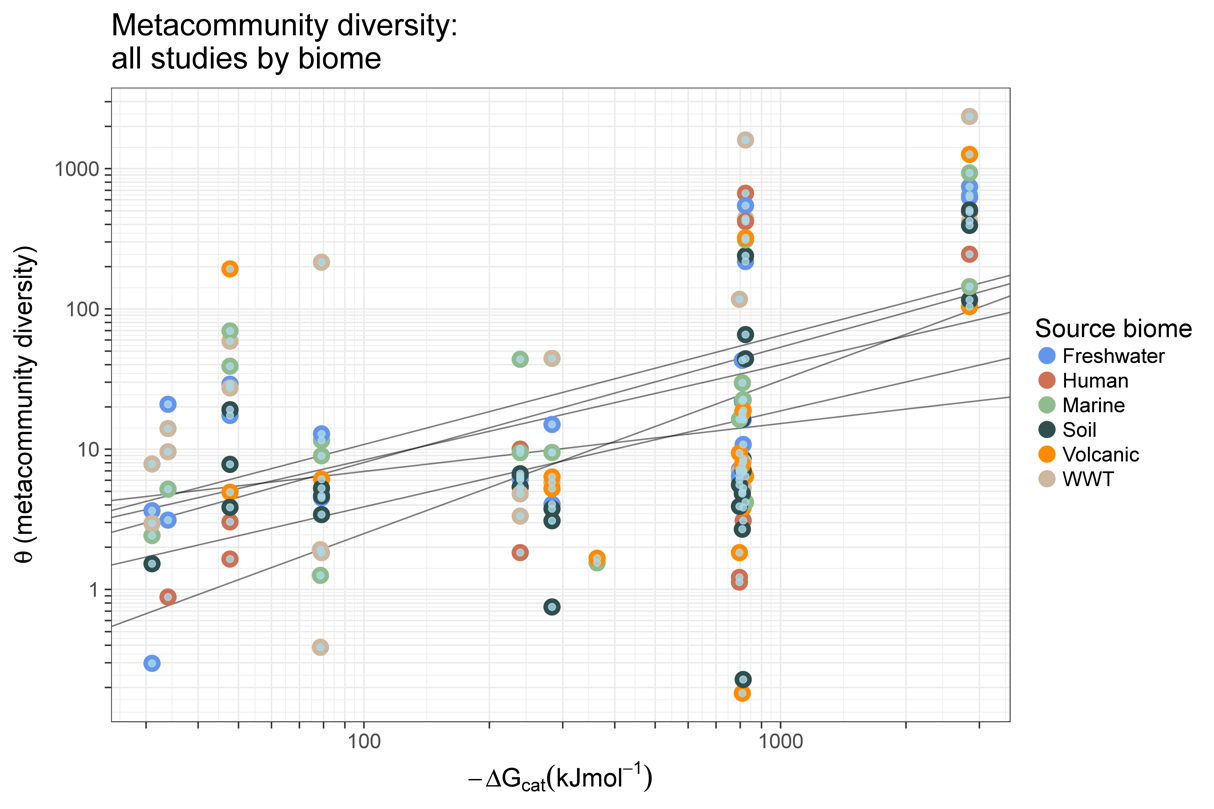


Figure S2. The relationship between the estimated the metacommunity diversity in all biomes and the natural log of the energetic yield (P slope <0.001; Adjusted R^2^ = 30%). With the natural log comparison the data was highly significant (P<0.001; adjusted R^2^ 29.6). Normal residuals were observed.


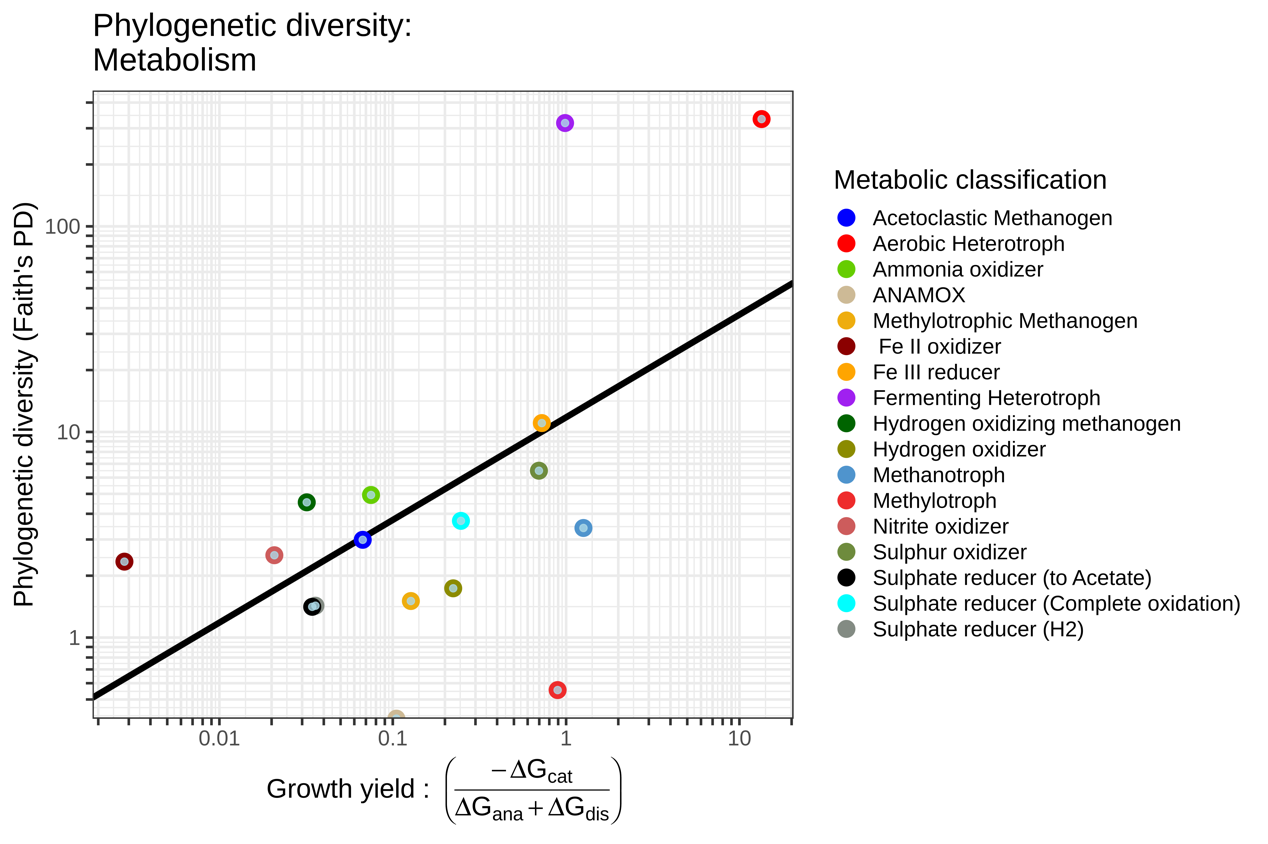


Figure S3. The relationship between Phylogenetic Distances within functional groups in the Greengenes database and estimated energetic yield under standard conditions (P value for the slope = 0.02 Adjusted R^2^ 29%). The relationship between Phylogenetic Distances between functional groups in the Greengenes database and the catabolic energy available to the cell (kJmol^-1^ of electron donor) under standard conditions (P value for the slope was 0.025; R^2^ Adjusted = 25%).  These results are shown as we committed ourselves to showing all data examined in the study. However, because this database is not a random sample of the diversity, we do not believe it is suitable for testing our hypothesis.


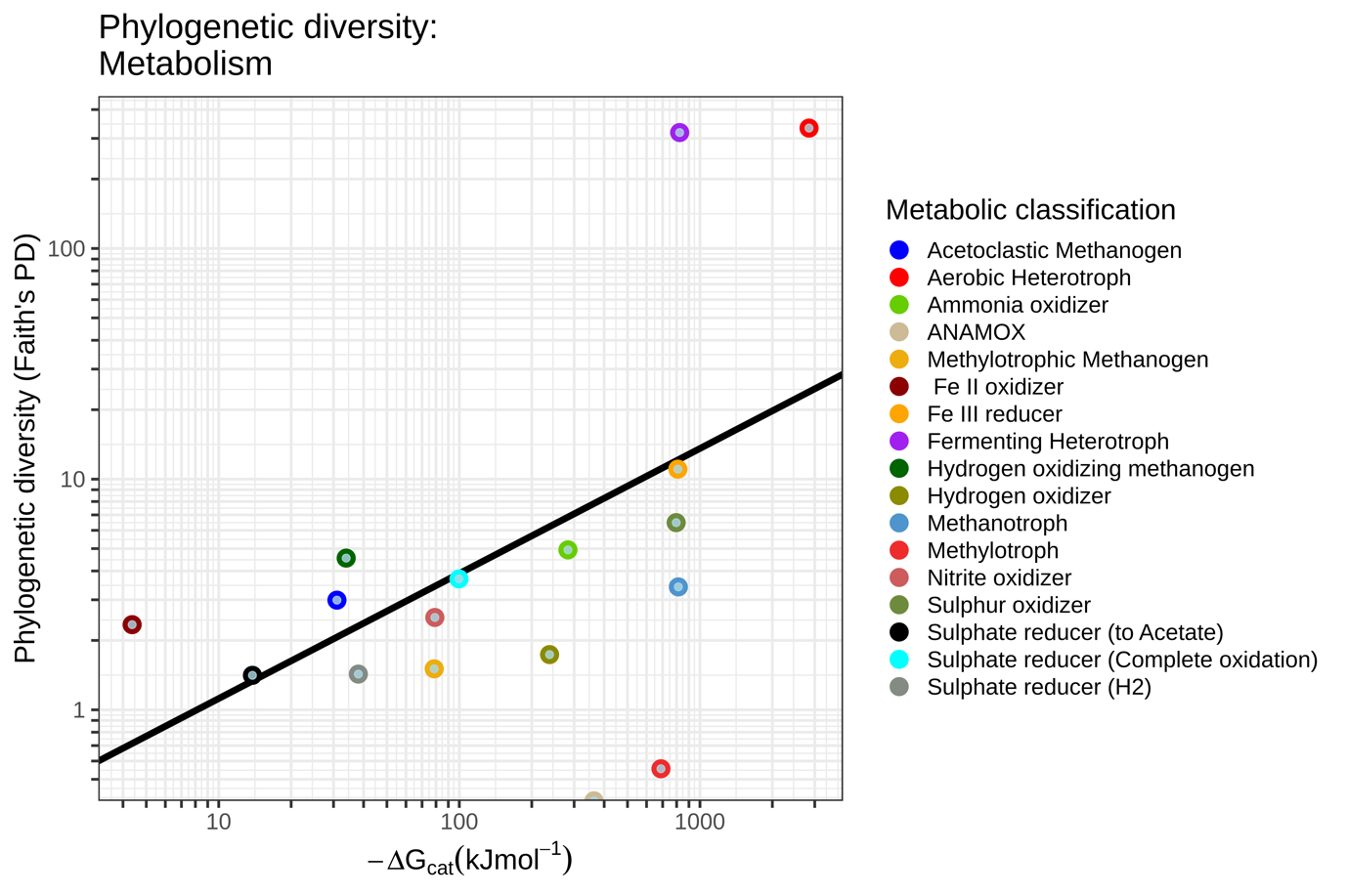


Fig. S4. The relationship between Phylogenetic Distances between functional groups in the Greengenes database and the catabolic energy available to the cell (kJmol^-1^ of electron donor) under standard conditions (P value for the slope was 0.025; R^2^ Adjusted = 25%).  These results are shown as we committed ourselves to showing all data examined in the study. However, because this database is not a random sample of the diversity, we do not believe it is suitable for testing the hypothesis.

References

DeLong JP, Okie JG, Moses ME *et al.* Shifts in metabolic scaling, production, and efficiency across major evolutionary transitions of life. Proceedings of the National Academy of Sciences of the United States of America 2010;**107**: 12941-5.

Makarieva AM, Gorshkov VG, Li BL. Energetics of the smallest: do bacteria breathe at the same rate as whales? Proceedings of the Royal Society B-Biological Sciences 2005;**272**: 2219-24.

Prochazk.Gj, Payne WJ, Mayberry WR. Calorific Content of Certain Bacteria and Fungi. . Journal of Bacteriology 1970;**104**: 646-&.
